# Deep learning feature-based model for predicting lymphovascular invasion in urothelial carcinoma of bladder using CT images

**DOI:** 10.1186/s13244-025-01988-6

**Published:** 2025-05-18

**Authors:** Bangxin Xiao, Yang Lv, Canjie Peng, Zongjie Wei, Qiao Xv, Fajin Lv, Qing Jiang, Huayun Liu, Feng Li, Yingjie Xv, Quanhao He, Mingzhao Xiao

**Affiliations:** 1 Department of Urology, The First Affiliated Hospital of Chongqing Medical University, Chongqing, China; 2 Department of Urology, Yongchuan Hospital of Chongqing Medical University, Chongqing, China; 3 Department of Radiology, The First Affiliated Hospital of Chongqing Medical University, Chongqing, China; 4 Department of Urology, The Second Affiliated Hospital of Chongqing Medical University, Chongqing, China; 5 Outpatient Department, The Second Affiliated Hospital, Army Medical University, Chongqing, China; 6 Department of Urology, Chongqing University Three Gorges Hospital, Chongqing, China

**Keywords:** Urothelial carcinoma of the bladder, Lymphovascular invasion, Deep learning model, Stacking learning model, Multicenter retrospective study

## Abstract

**Objectives:**

Lymphovascular invasion significantly impacts the prognosis of urothelial carcinoma of the bladder. Traditional lymphovascular invasion detection methods are time-consuming and costly. This study aims to develop a deep learning-based model to preoperatively predict lymphovascular invasion status in urothelial carcinoma of bladder using CT images.

**Methods:**

Data and CT images of 577 patients across four medical centers were retrospectively collected. The largest tumor slices from the transverse, coronal, and sagittal planes were selected and used to train CNN models (InceptionV3, DenseNet121, ResNet18, ResNet34, ResNet50, and VGG11). Deep learning features were extracted and visualized using Grad-CAM. Principal Component Analysis reduced features to 64. Using the extracted features, Decision Tree, XGBoost, and LightGBM models were trained with 5-fold cross-validation and ensembled in a stacking model. Clinical risk factors were identified through logistic regression analyses and combined with DL scores to enhance lymphovascular invasion prediction accuracy.

**Results:**

The ResNet50-based model achieved an AUC of 0.818 in the validation set and 0.708 in the testing set. The combined model showed an AUC of 0.794 in the validation set and 0.767 in the testing set, demonstrating robust performance across diverse data.

**Conclusion:**

We developed a robust radiomics model based on deep learning features from CT images to preoperatively predict lymphovascular invasion status in urothelial carcinoma of the bladder. This model offers a non-invasive, cost-effective tool to assist clinicians in personalized treatment planning.

**Critical relevance statement:**

We developed a robust radiomics model based on deep learning features from CT images to preoperatively predict lymphovascular invasion status in urothelial carcinoma of the bladder.

**Key Points:**

- We developed a deep learning feature-based stacking model to predict lymphovascular invasion in urothelial carcinoma of the bladder patients using CT.
- Max cross sections from three dimensions of the CT image are used to train the CNN model.
- We made comparisons across six CNN networks, including ResNet50.

**Graphical Abstract:**

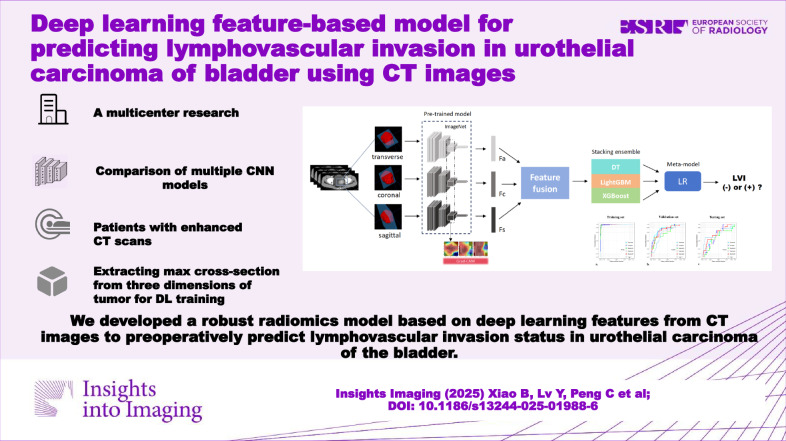

## Introduction

Bladder cancer is the tenth most common cancer worldwide, with approximately 573,000 new cases and 213,000 deaths annually [1]. Urothelial carcinoma accounts for about 95% of bladder cancers [2]. Lymphovascular invasion (LVI) is closely associated with the prognosis of urothelial carcinoma of the bladder (UCB). Several studies have identified LVI as an independent predictor of bladder cancer recurrence, cancer-specific survival, and overall survival [3–5]. The presence of LVI in tumors suggests a higher invasive potential [6, 7].

Pathological reports of bladder cancer should include LVI status: absent, present, or indeterminate [8]. Identifying LVI accurately with routine Hematoxylin and Eosin (HE) staining is challenging. The gold standard is Immunohistochemistry (IHC) staining using endothelial markers like CD31 and CD34 [9]. However, IHC staining is time-consuming and costly [8], making it impractical in time-sensitive situations and economically disadvantaged regions. Thus, developing an AI model based on preoperative imaging for rapid LVI diagnosis is highly desirable.

With the advancement of technology, AI has found extensive applications in medicine with promising prospects [10, 11]. Radiomics, a major area of AI application, provides clinicians with features in medical images that may not be observable by the naked eye, thus helping them to more accurately diagnose diseases and determine prognosis [12, 13]. Several studies have applied radiomics to predict LVI status in various tumors [14–17]. However, to the best of our knowledge, there is still no CT-based model for predicting the LVI status of UCB.

Traditional radiomics methods extract a large number of quantitative features from images through classical image processing techniques such as filtering, texture analysis, and morphological analysis. These features include shape, edges, texture, and intensity distribution. Traditional methods generally rely on manually extracting features, where researchers must select appropriate features based on their experience [12, 18]. This process is not only time-consuming but also susceptible to subjective influences, potentially missing some important features. In contrast, deep learning, especially convolutional neural networks (CNNs), can automatically learn and extract features from image data. It can identify complex patterns that are difficult to capture manually, thus improving the efficiency and accuracy of feature extraction [19, 20]. The features learned automatically are typically more representative and robust than manually designed features, enabling better capture of complex patterns in images. Therefore, deep learning offers higher predictive accuracy and reliability in many application scenarios.

Considering the above factors, we developed a model based on deep learning features using preoperative CT images to predict postoperative pathological LVI status of UCB patients.

## Methods

### Patients

**Fig. 1:**
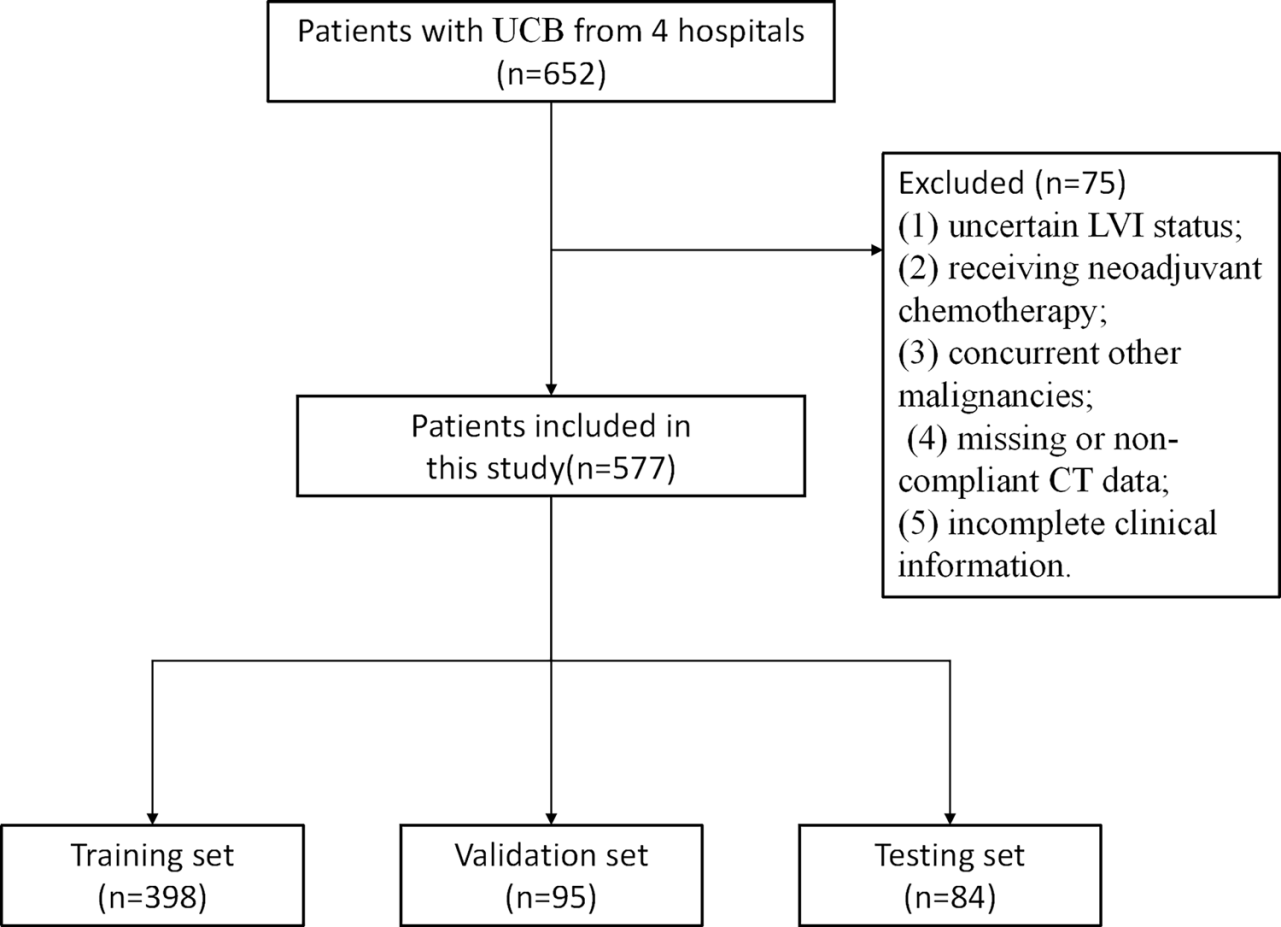
The detailed flowchart of the patient recruitment process. UCB, urothelial carcinoma of the bladder; LVI, lymphovascular invasion

This study was ethically approved (K2024-187-01) with informed consent waived. This study follows the CLEAR checklist guidelines [21]. We retrospectively collected data of UCB patients undergoing radical cystectomy and pathologically confirmed from four medical institutions. The study period spanned from 2013 to 2023 for Center 1 (First Affiliated Hospital of Chongqing Medical University), 2016 to 2023 for Center 2 (Yongchuan Hospital of Chongqing Medical University), 2013 to 2023 for Center 3 (Second Affiliated Hospital of Chongqing Medical University), and 2013 to 2022 for Center 4 (Chongqing University Three Gorges Hospital). Inclusion criteria were: (1) pathologically confirmed urothelial carcinoma, (2) radical cystectomy performed, and (3) enhanced CT scan within 20 days preoperatively. Exclusion criteria were: (1) uncertain LVI status; (2) receiving neoadjuvant chemotherapy; (3) concurrent other malignancies; (4) missing or non-compliant CT data; (5) incomplete clinical information. A total of 577 patients were included, with participants from Center 1 (*n* = 398) in the training set, Center 2 (*n* = 39) and Center 3 (*n* = 56) in the validation set, and Center 4 (*n* = 84) in the testing set. The inclusion and exclusion flowchart is shown in Fig. 1.

### Clinical and pathological data

Clinical and pathological data, including age, gender, pathological TNM stage, and pathological grade, were collected from electronic medical record systems. Pathological tumor staging was based on the American Joint Committee on Cancer Staging Manual (8th edition). The LVI status of all UCB patients was confirmed by the pathological reports.

### Image collection

CT scan images were downloaded from the Picture Archiving and Communication System (PACS) and saved in the original Digital Imaging and Communications in Medicine (DICOM) format. The nephrographic phase (NP) CT images were collected for subsequent analysis. Detailed CT imaging parameters from different centers are provided in Supplementary Table S1.

### Patient and CT morphological characteristics

Basic information of all patients was recorded. Two radiologists independently and retrospectively interpreted all CT images included in this study and were blinded to other patient information. Differences are resolved through discussion. Recorded characteristics included (largest one in case of multiple tumors): (1) maximum diameter (cm), (2) number of tumors (single or multiple), (3) tumor margin (well-defined or blurred), (4) tumor location (left wall, right wall, anterior wall, posterior wall, parietal wall, bladder neck), (5) tumor growth style (within bladder wall or into bladder cavity), and (6) calcification (present or absent).

### Image preprocessing and region of interest segmentation

CT images were standardized with a window level of 30 and a window width of 250. CT images were resampled to a voxel size of 0.7 × 0.7 × 0.7 mm using the linear interpolation method. Two experienced radiologists delineated the region of interest (ROI) along the edges of cancer of bladder on CT images using ITK-SNAP software (v.4.0.0; http://www.itksnap.org). Discrepancies in ROI delineation were resolved by discussion between the two radiologists.

### Deep learning model construction

The largest tumor slice from the transverse, coronal, and sagittal planes of the 3D mask was selected, respectively, removing background and retaining only the tumor mask region. All transverse, coronal, and sagittal slices from the training set were input into CNN models for training, respectively. The model for each dimension was optimized the network parameters separately as an independent branch during the training process.

The deep learning models used for transfer learning in this study include InceptionV3, DenseNet121, ResNet18, ResNet34, ResNet50, and VGG11. These models were pre-trained on the ImageNet dataset to obtain initial weights. For model training, the input section for InceptionV3 was adjusted to 299 × 299 pixels, while for other CNN models, it was 224 × 224 pixels, and all images were z-score normalized. Data enhancement strategies were applied, involving randomized horizontal and vertical flipping. During training, the parameters of CNN models are iteratively updated by backpropagation, and the cross-entropy loss function is employed. The learning rate was set to 0.01, and the Stochastic Gradient Descent (SGD) optimizer was used to update the parameters. Batch size was set to 64, and L2 regularization and early stopping strategies were employed to prevent overfitting. Training was conducted on an NVIDIA 4090D (24G). Detailed model training process is shown in the Supplementary materials.

After transfer learning training of the deep learning models, deep learning features were extracted from the last average pooling layer of the model. Gradient-weighted class activation mapping (Grad-CAM) was used for model visualization to make the model’s decision process more transparent and study its interpretability. Gradients from the final convolutional layer of the CNN were used for weighted fusion, obtaining a class activation map highlighting important regions for classification.

**Fig. 2:**
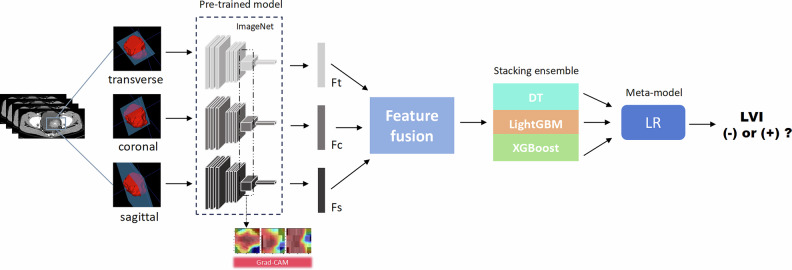
Workflow diagram for the development of the predictive models. The largest tumor slices from the transverse, coronal, and sagittal planes were selected and used to train CNN models (InceptionV3, DenseNet121, ResNet18, ResNet34, ResNet50, and VGG11). Ft, features extracted from transversal; Fc, features extracted from coronal; Fs, features extracted from sagittal

The features extracted from the models of the three planes were fused by calculating the mean. The fusion features were then processed in the following steps. First, Principal Component Analysis (PCA) was applied to the training set to reduce the feature dimensionality to 64 components. The same PCA transformation was subsequently applied to the validation and testing sets, ensuring consistent dimensionality reduction across all datasets. Second, Synthetic Minority Over-sampling Technique (SMOTE) is performed on the training set to address the class imbalance [22]. Third, the features were normalized. Fourth, we applied LASSO to further select features. Finally, we use Pearson correlation analysis (threshold = 0.8) to remove highly correlated features. Based on the selected features, Decision Tree, XGBoost, and LightGBM models were constructed on the training set using 5-fold cross-validation. Stacking ensemble learning technique was employed, with predictions from the three models in each fold as input features for the meta-model (Logistic Regression), resulting in a final stacking ensemble model. The detailed model construction process is shown in Fig. 2.

### Clinical model and combined model construction

Univariate logistic regression analysis was used to select clinical characteristics with *p* < 0.05. Multivariate logistic regression analysis was then performed on the clinical characteristics identified by univariate analysis to determine potential LVI risk factors in the clinical data, with *p* < 0.05 considered statistically significant. Based on the above LVI clinical risk factors, a clinical model was developed using logistic regression algorithm. A combined model was developed using the clinical risk factors and stacking model predictions (DL scores) through logistic regression algorithm.

### Statistical methods

The performance of the models was evaluated using area under the curve (AUC), accuracy (ACC), sensitivity, and specificity. Decision curve analysis was used to measure the clinical utility of the models. Statistical analysis was conducted using SPSS (version 25.0) and R (version 4.4.0). Continuous and categorical variables in baseline analysis were analyzed using one-way ANOVA and Chi-square tests, respectively. Delong test was used to compare AUCs, with *p* < 0.05 indicating statistical significance. Receiver operating characteristic curves were plotted, and the Youden index (Youden index = sensitivity + specificity − 1) was calculated to determine the optimal cutoff point for DL scores, where the maximum Youden index was the optimal threshold.

## Results

### Patient characteristics

Baseline clinical characteristics of the patients are shown in Table 1. Among the 577 patients included in the study, ages ranged from 30 to 90 years (mean 66.08 years), with 509 (88.2%) males and 68 (11.8%) females. Clinical characteristics were similar between the groups. Among all patients, 87 (15.1%) were LVI-positive. In the training set, 50 (12.6%) were LVI-positive. In the validation set, 22 (23.2%) were LVI-positive, and in the testing set, 15 (17.9%) were LVI-positive.

**Table 1:** The baseline clinical characteristics of patients in three sets

Characteristic	All patients (*n* = 577)	Training dataset (*n* = 398)	Validation dataset (*n* = 95)	Test dataset (*n* = 84)	*p*-value
Age (years)					0.363
≤ 60	157 (27.2%)	105 (26.4%)	23 (24.2%)	29 (34.5%)	
61–70	219 (38.0%)	147 (36.9%)	41 (43.2%)	31 (36.9%)	
> 70	201 (34.8%)	146 (36.7%)	31 (32.6%)	24 (28.6%)	
Gender					0.421
Male	509 (88.2%)	354 (88.9%)	80 (84.2%)	75 (89.3%)	
Female	68 (11.8%)	44 (11.1%)	15 (15.8%)	9 (10.7%)	
Number of tumors					0.037
Single	447 (77.5%)	303 (76.1%)	70 (73.7%)	74 (88.1%)	
Multiple	130 (22.5%)	95 (23.9%)	25 (26.3%)	10 (11.9%)	
pT					< 0.01
Ta	48 (8.3%)	38 (9.5%)	10 (10.5%)	0 (0.0%)	
1	155 (26.9%)	126 (31.7%)	29 (30.5%)	0 (0.0%)	
2	231 (40.0%)	130 (32.7%)	26 (27.4%)	75 (89.3%)	
3	100 (17.3%)	75 (18.8%)	19 (20.0%)	6 (7.1%)	
4	43 (7.5%)	29 (7.3%)	11 (11.6%)	3 (3.6%)	
pN					0.249
0	538 (93.2%)	373 (93.7%)	85 (89.5%)	80 (95.2%)	
1	16 (2.8%)	12 (3.0%)	2 (2.1%)	2 (2.4%)	
2	23 (4.0%)	13 (3.3%)	8 (8.4%)	2 (2.4%)	
pM					0.228
0	573 (99.3%)	396 (99.5%)	93 (97.9%)	84 (100.0%)	
1	4 (0.7%)	2 (0.5%)	2 (2.1%)	0 (0.0%)	
AJCC					< 0.001
0a	49 (8.5%)	39 (9.8%)	10 (10.5%)	0 (0.0%)	
I	153 (26.5%)	125 (31.4%)	28 (29.5%)	0 (0.0%)	
II	223 (38.6%)	127 (31.9%)	24 (25.3%)	72 (85.7%)	
III	148 (25.6%)	105 (26.4%)	31 (32.6%)	12 (14.3%)	
IV	4 (0.7%)	2 (0.5%)	2 (2.1%)	0 (0.0%)	
Grade					0.018
0	79 (13.7%)	51 (12.8%)	21 (22.1%)	7 (8.3%)	
1	498 (86.3%)	347 (87.2%)	74 (77.9%)	77 (91.7%)	
Diameter (cm)					0.794
< 3.5	293 (50.8%)	202 (50.8%)	46 (48.4%)	45 (53.6%)	
≥ 3.5	284 (49.2%)	196 (49.2%)	49 (51.6%)	39 (46.4%)	
Location					0.003
Left wall	124 (21.5%)	90 (22.6%)	17 (17.9%)	17 (20.2%)	
Right wall	94 (16.3%)	55 (13.8%)	13 (13.7%)	26 (31.0%)	
Anterior wall	72 (12.5%)	47 (11.8%)	14 (14.7%)	11 (13.1%)	
Posterior wall	259 (44.9%)	187 (47.0%)	44 (46.3%)	28 (33.3%)	
Parietal wall	9 (1.6%)	3 (0.8%)	4 (4.2%)	2 (2.4%)	
Bladder neck	19 (3.3%)	16 (4.0%)	3 (3.2%)	0 (0.0%)	
Calcification					0.674
Absent	475 (82.3%)	326 (81.9%)	77 (81.1%)	72 (85.7%)	
Present	102 (17.7%)	72 (18.1%)	18 (18.9%)	12 (14.3%)	
Boundary					0.775
Well-defined	360 (62.4%)	245 (61.6%)	60 (63.2%)	55 (65.5%)	
Blurred	217 (37.6%)	153 (38.4%)	35 (36.8%)	29 (34.5%)	
Tumor growth style					0.031
Within wall	116 (20.1%)	82 (20.6%)	25 (26.3%)	9 (10.7%)	
Into cavity	461 (79.9%)	316 (79.4%)	70 (73.7%)	75 (89.3%)	
LVI					0.027
Negative	490 (84.9%)	348 (87.4%)	73 (76.8%)	69 (82.1%)	
Positive	87 (15.1%)	50 (12.6%)	22 (23.2%)	15 (17.9%)	

*pT* pathological T stage, *pN* pathological N stage, *AJCC* American Joint Committee on Cancer, *LVI* Lymphovascular invasion

### Deep learning model performance

**Fig. 3:**
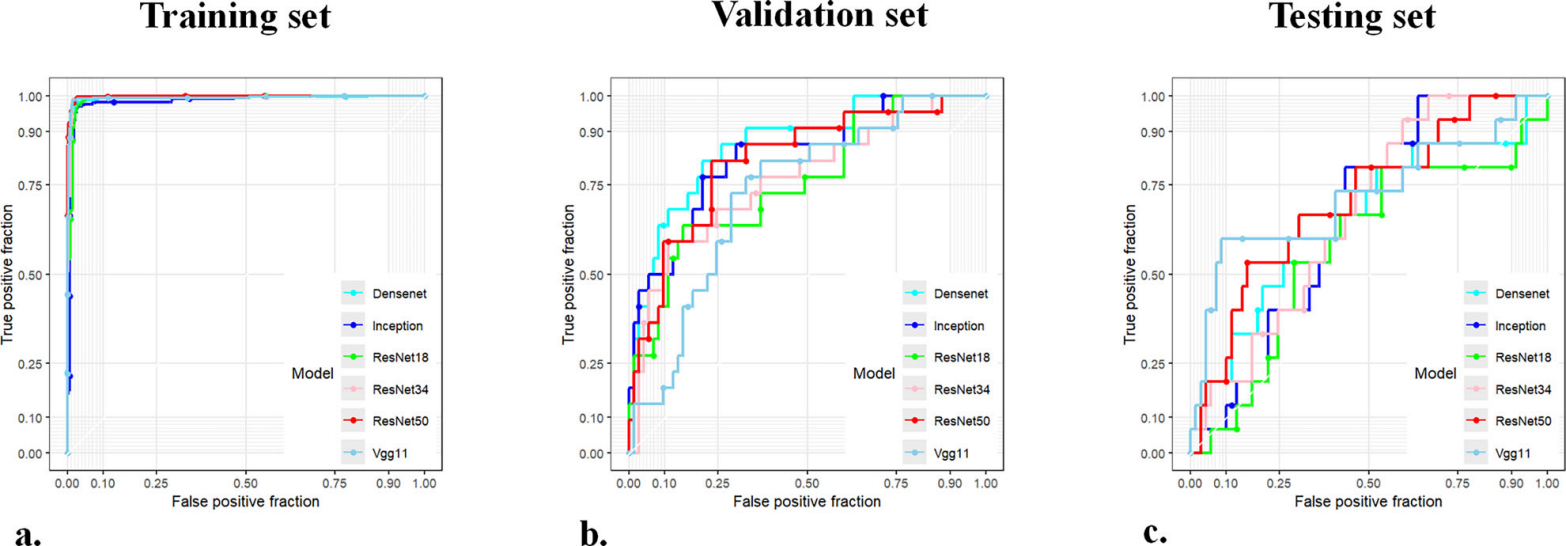
Receiver operating characteristic curves of different deep learning models in the (**a**) training set, (**b**) validation set and (**c**) testing sets, respectively

After image preprocessing and data augmentation, a total of 2925 images were included in this study. Among these, 2388 images were allocated for model training, 285 images were used for model validation, and 252 images were reserved for external testing to evaluate the model’s generalizability. We trained several ensemble models based on deep learning features to predict the LVI status of UCB using preoperative CT images. The overall performance parameters of the ResNet50 deep learning model, trained through transfer learning, were superior to other deep learning models. In the validation set, the stacking machine learning model based on the ResNet50 network had an AUC of 0.818 (0.714, 0.922), ACC of 0.779 (0.775, 0.782), sensitivity of 0.818, and specificity of 0.767. In the testing set, the AUC was 0.708 (0.563, 0.853), ACC was 0.786 (0.782, 0.790), sensitivity was 0.533, and specificity was 0.841. Performance of each model is shown in Table 2 and Fig. 3.

**Table 2:** The performance comparison of CNN models

Model	Set	AUC	95% CI	ACC	95% CI	SEN	SPE
Densenet121	Train	0.992	0.985, 0.999	0.980	0.980, 0.980	0.977	0.983
	Val	0.865	0.778, 0.952	0.800	0.797, 0.803	0.818	0.795
	Test	0.651	0.484, 0.818	0.536	0.530, 0.542	0.800	0.478
Inception_V3	Train	0.986	0.977, 0.995	0.971	0.971, 0.971	0.971	0.971
	Val	0.833	0.733, 0.932	0.789	0.786, 0.793	0.773	0.795
	Test	0.664	0.537, 0.790	0.607	0.602, 0.613	0.800	0.565
ResNet18	Train	0.993	0.987, 0.998	0.973	0.973, 0.973	0.983	0.963
	Val	0.758	0.639, 0.877	0.800	0.797, 0.803	0.636	0.849
	Test	0.575	0.407, 0.743	0.524	0.518, 0.530	0.800	0.464
ResNet34	Train	0.994	0.989, 1.000	0.989	0.988, 0.989	0.991	0.986
	Val	0.765	0.643, 0.886	0.821	0.818, 0.824	0.591	0.890
	Test	0.671	0.539, 0.802	0.500	0.494, 0.506	0.933	0.406
ResNet50	Train	0.999	0.997, 1.000	0.986	0.986, 0.986	0.997	0.974
	Val	0.818	0.714, 0.922	0.779	0.775, 0.782	0.818	0.767
	Test	0.708	0.563, 0.853	0.786	0.782, 0.790	0.533	0.841
Vgg11	Train	0.995	0.989, 1.000	0.987	0.987, 0.987	0.989	0.986
	Val	0.729	0.615, 0.842	0.674	0.669, 0.678	0.818	0.630
	Test	0.719	0.547, 0.890	0.857	0.854, 0.860	0.600	0.913

*CNN* convolutional neural network, *AUC* area under the curve, *ACC* accuracy, *SEN* sensitivity, *SPE* specificity, *95% CI* confidence interval = 95%, *Train* training set, *Val* validation set, *Test* testing set

**Fig. 4:**
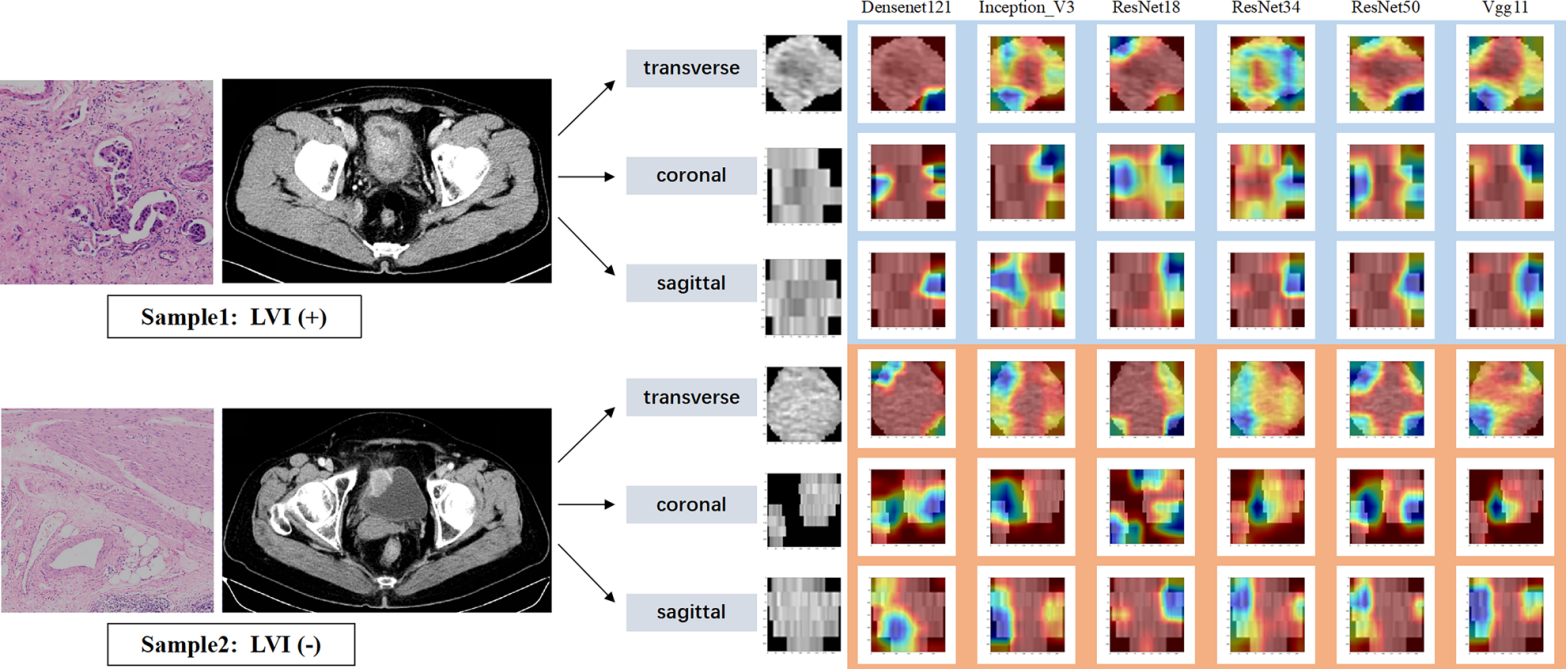
The attention regions of all deep learning models in LVI-positive and negative samples from three dimensions of CT images

Grad-CAMs of all deep learning models are shown in Fig. 4, indicating that the deep learning models focused mainly on the tumor boundaries and internal regions and the attention region of ResNet50 is more reasonable and superior.

### Clinical model and combined model performance

Univariate and multivariate logistic regression analyses were performed on clinical characteristics. Pathologic T-staging, pathologic N-staging, tumor location, histological grade, and tumor growth style were significantly associated with LVI (*p* < 0.05), as shown in Table 3. Among these factors, pathologic T-staging, tumor location, and tumor growth style were identified as independent risk factors for LVI.

**Table 3:** Univariate logistic regression analysis and multivariate logistic regression analysis of clinical characteristics

Variable	Univariate		Multivariate	
	OR	*p*-value	OR	*p*-value
Age	1.154 (0.789, 1.689)	0.460		
Gender	3.294 (0.772, 14.054)	0.107		
Number of tumors	1.128 (0.553, 2.301)	0.740		
pT	2.951 (2.116, 4.116)	**< 0.001**	2.698 (1.887, 3.859)	**< 0.001**
pN	2.643 (1.546, 4.519)	**< 0.001**	1.375 (0.766, 2.468)	0.285
Grade	8.221 (1.110, 60.894)	**0.039**	3.590 (0.463, 27.824)	0.221
Diameter	1.497 (0.822, 2.726)	0.187		
Location	1.341 (1.067, 1.686)	**0.012**	1.375 (1.073, 1.762)	**0.012**
Calcification	0.583 (0.238, 1.425)	0.236		
Boundary	0.888 (0.479, 1.644)	0.704		
Tumor growth style	0.446 (0.234, 0.849)*	**0.014**	0.464 (0.223, 0.964)*	**0.040**

The variables with *p* < 0.05 in the univariable logistic regression analysis were enrolled into multivariable logistic regression analysis, and *p*-value < 0.05 is shown in bold

*OR* odds ratio, *pT* pathological T stage, *pN* pathological N stage

* Reference = ‘within bladder wall’

A clinical model was developed based on clinical risk factors using logistic regression algorithm. In the validation and testing set, the AUCs of the clinical model were 0.776 (0.667, 0.875) and 0.665 (0.471, 0.855). More detailed model performance is shown in Supplementary Table S5.

**Fig. 5:**
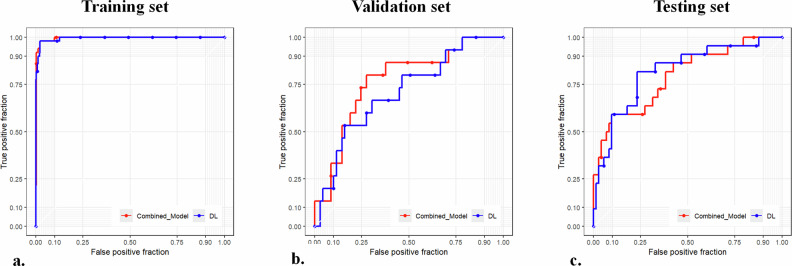
Receiver operating characteristic curves of the DL model and the combined model in the (**a**) training set, (**b**) validation set and (**c**) testing sets, respectively. DL, the DL model (the ResNet50-based stacking model)

A combined model was constructed based on clinical risk factors and DL scores using a logistic regression algorithm. In the validation set, the combined model had an AUC of 0.794 (0.682, 0.906), ACC of 0.832 (0.829, 0.834), sensitivity of 0.591, and specificity of 0.904. In the testing set, the AUC was 0.767 (0.635, 0.899), ACC was 0.738 (0.734, 0.743), sensitivity was 0.800, and specificity was 0.725. Performance of the models is shown in Table 4 and Fig. 5.

**Table 4:** The performance comparison of DL model and the combined model

	Model	AUC	*p*-value	95% CI	ACC	95% CI	SEN	SPE
Train	DL	0.999	refer	0.997, 1.000	0.986	0.986, 0.986	0.997	0.974
	Combined	0.995	0.952	0.989, 1.000	0.982	0.967, 0.992	0.940	0.989
Val	DL	0.818	refer	0.714, 0.922	0.779	0.775, 0.782	0.818	0.767
	Combined	0.794	0.595	0.682, 0.906	0.832	0.829, 0.834	0.591	0.904
Test	DL	0.708	refer	0.563, 0.853	0.786	0.782, 0.790	0.533	0.841
	Combined	0.767	0.316	0.635, 0.899	0.738	0.734, 0.743	0.800	0.725

*AUC* area under the curve, *ACC* accuracy, *SEN* sensitivity, *SPE* specificity, *95% CI* confidence interval = 95%, *Train* training set, *Val* validation set, *Test* testing set

## Discussion

The presence of LVI significantly impacts the prognosis of cancer patients. Numerous studies have shown that LVI is an independent prognostic factor and biomarker for bladder tumors [23–25]. Additionally, the significance of LVI has been studied in various types of cancer; LVI independently influences the locoregional recurrence and metastasis of low-stage cervical squamous cell carcinomas and disease-specific mortality for all tumors [26]; LVI plays a pivotal role in the prognostic prediction of superficial esophageal cancer [27]; LVI presence is a prognostic marker in patients with cervical squamous cell carcinoma [28]; LVI has been identified as an independent prognostic factor in gastric cancer patients [29].

In this multicenter study, we proposed an ensemble learning model based on deep learning features to predict the LVI status in patients with UCB using preoperative CT images. Compared to other models, the ResNet50 deep learning model constructed in this study demonstrated good discriminatory ability for LVI status in UCB. Although some clinical characteristics in the baseline analysis had statistical differences between the three groups, likely due to demographic differences, the DL model remained stable across diverse data, further proving its robustness (AUC = 0.818 [0.714, 0.922]; 0.708 [0.563, 0.853]). In comparison with the clinical model (Table S5), the DL model showed better performance and stronger generalization ability in both the validation set and the testing set.

Among the six DL models, the performance ranking of the models follows the pattern: ResNet50 > ResNet34 > ResNet18, indicating a gradual improvement in performance with increased model depth. The ResNet models (ResNet18, ResNet34, and ResNet50) incorporate Residual Blocks with skip connections, which alleviate the gradient vanishing problem in deeper networks. With increased depth, the model gains the capacity to learn more complex and fine-grained features. Consequently, ResNet50, having more layers than ResNet34 and ResNet18, captures a greater variety of data patterns, enhancing generalization and achieving superior performance. As shown in Fig. 4, the models primarily focus on the boundary and interior of the tumor; occasionally, regions of high attention (red) appear in the surrounding black background, suggesting potential misalignment in the model’s attention. However, no model can guarantee that its attention will exclusively align with the target region in any case.

After applying SMOTE, the performance of the deep learning model generally decreased (Table S3), which we believe is a result of eliminating the bias caused by class imbalance. However, after SMOTE processing, the model’s calibration curve became closer to the diagonal (Figs. S3, S5), indicating a positive effect of SMOTE on model calibration. With SMOTE applied, the model is more clinically reliable and shows better clinical utility.

Radiomics can extract and analyze high-throughput quantitative data from medical images, providing non-invasive tools for identifying tumor biological behaviors. In recent years, more radiomics models based on CT or MRI have been used to predict LVI status in tumor patients. Du et al [15] developed an ultrasound-based radiomics model for preoperative prediction of lymphovascular invasion in patients with invasive breast cancer. Liu et al [16] developed a deep learning model for diagnosing LVI and predicting prognosis in clinically staged lung adenocarcinoma patients. Wang et al [30] established a machine learning model based on enhanced CT to effectively predict preoperative LVI status in patients with esophageal squamous cell carcinoma. These studies have shown that it is feasible to predict tumor LVI status from preoperative imaging and clinical characteristics. Similarly, in this study, the combined model achieved high performance in predicting LVI status in UCB (AUC = 0.794 [0.682, 0.906]; 0.767 [0.635, 0.899]). The combined model demonstrated slightly lower performance than ResNet50 on both the training and validation sets, though this difference was not statistically significant. However, on the testing set, the combined model outperformed the deep learning model, likely due to the stability and high performance of the DL score developed in this study. Accordingly, we consider the combined model to be both superior and more robust than the deep learning model alone. Thus, the combined model, which incorporates clinical factors, is selected as the final model for this study.

Previously, Ceachi et al [9] developed an AI model based on pathological slides of urothelial carcinoma for LVI diagnosis, but it was a single-center study with unverified generalization capability. However, this study, involving four independent centers, validated the model’s robustness and generalization ability. Moreover, our model’s input was preoperative CT images, eliminating potential diagnostic interferences from surgical and specimen preparation processes.

The gold standard for LVI pathological detection is IHC staining using endothelial markers, but this method is expensive and time-consuming. IHC staining is not available for intraoperative frozen section examinations and in economically disadvantaged regions where not all patients can afford this examination. In these cases, our deep learning model provides a timely and cost-effective decision support tool for clinicians.

Although there is no direct evidence that currently proves LVI’s role in lymph node metastasis, existing research generally agrees on a strong correlation between LVI and lymph node metastasis in UCB [31]. Some studies have demonstrated that LVI is an independent risk factor for lymph node metastasis in UCB [32, 33], while others indicate LVI’s independent prognostic significance in lymph node-negative patients [5, 34–36]. The preliminary results of this study indicate a correlation between LVI and lymph node metastasis in UCB (pathologic N-staging in univariate logistic regression, *p* < 0.05). The DL model can assist surgeons in predicting LVI status preoperatively. When the model predicts a positive result, it can guide surgeons to perform more detailed and extensive lymph node dissection in radical cystectomy [31]. The combined model combines postoperative clinical characteristics with the DL model to provide a more accurate diagnosis, guiding doctors in intravesical instillation and chemotherapy management when necessary. As a non-invasive examination tool, the DL model and the combined model can play multiple roles in preoperative surgical planning and postoperative chemotherapy management, significantly assisting doctors in managing UCB with LVI.

Our study has some limitations. First, it is a retrospective study, inevitably introducing selection bias and hidden confounding factors. Second, this study included four independent institutions; however, the regions were limited, and the model’s generalization capability needs further validation on other regional datasets.

In conclusion, we have developed a stacking ensemble model based on deep learning features that can preoperatively determine the LVI status in UCB patients using CT images. This model provides a non-invasive, cost-effective tool that can aid clinicians in developing personalized healthcare strategies for patients.

## Supplementary information

ELECTRONIC SUPPLEMENTARY MATERIAL

## Data Availability

The datasets analyzed during the current study are not publicly available due to the need for follow-up research but are available from the corresponding author on reasonable request.

## Abbreviations

ACC: Accuracy
AUC: Area under the curve
CNN: Convolutional neural network
CT: Computed tomography
Grad-CAM: Gradient-weighted class activation mapping
IHC: Immunohistochemistry staining
LVI: Lymphovascular invasion
ROI: Region of interest
SMOTE: Synthetic minority over-sampling technique
UCB: Urothelial carcinoma of the bladder
