# Supplementary material for: Deep learning feature-based model for predicting lymphovascular invasion in urothelial carcinoma of bladder using CT images
Source: Insights Imaging. 2025 May 18;16:108. doi: 10.1186/s13244-025-01988-6 (PMC12086130; doi:10.1186/s13244-025-01988-6)
Supplement: Supplementary file 1 — ELECTRONIC SUPPLEMENTARY MATERIAL [file 13244_2025_1988_MOESM1_ESM.pdf]

Deep Learning Feature-based Model for Predicting Lymphovascular invasion in Urothelial Carcinoma of Bladder Using CT Images

ELECTRONIC SUPPLEMENTARY MATERIAL

Table S1 The CT protocols of the four centers

Parameters	Center 1	Center 2	Center 3	Center 4
CT version	Discovery CT750 HD (GE Healthcare, USA) or SOMATOM Definition Flash (Siemens Healthcare, Germany)	Brilliance iCT (Philips Healthcare, Netherlands)	Aquilion One (Toshiba Medical Systems, Japan); LightSpeed VCT (GE Healthcare, USA)	SOMATOM Definition AS (Siemens Healthcare, Germany); Revolution CT (GE Healthcare, USA)
CT tube voltage	100-120 kV	100–120 kV	110-120 kV	100-120 kV
CT tube current	200-500 mA	125–360 mAs	250-450 mA	100-250 mA
Gantry rotation time	0.50-0.60 s	0.27s	0.50s	0.28-0.33s
Detector collimation (mm)	0.625 mm	0.625 mm	0.625 mm	0.6-0.625 mm
Image matrix	512*512	512*512	512*512	512*512
Slice thickness	5-7 mm	1-7 mm	1-7 mm	1-5 mm

kV = kilovolt, mA = milliampere, mm = milimetre.

Introduction for CNN model training

```
{   batch_balance=True,
    normalize_method='imagenet',
    model_name='vgg11',
    gpus=[0],
    batch_size=64,
    epochs=100,
    init_lr=0.01,
    optimizer='sgd',
    retrain=None,
    iters_start=0,
    iters_verbose=1,
    pretrained=True   }
```

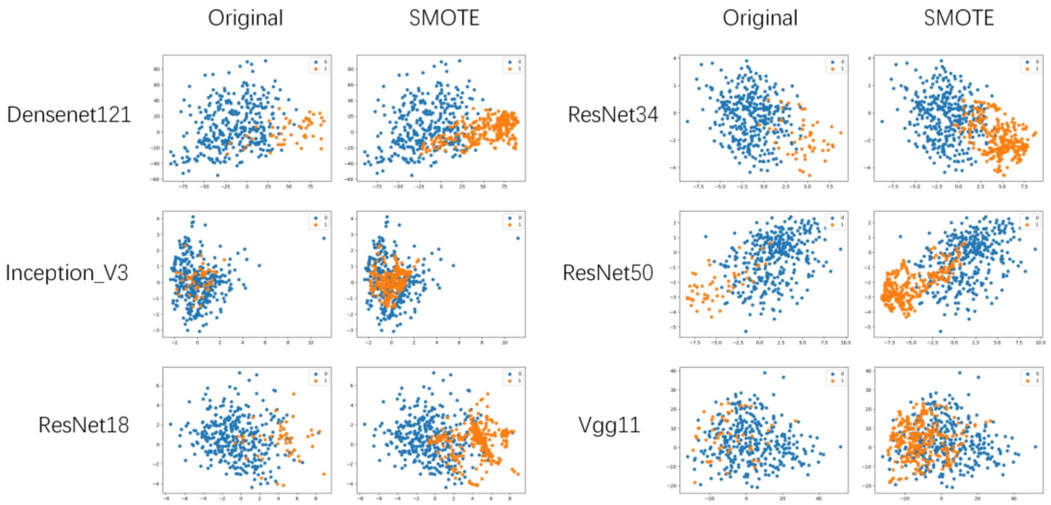

Figure S1 The distribution of original samples and distribution of SMOTE samples.

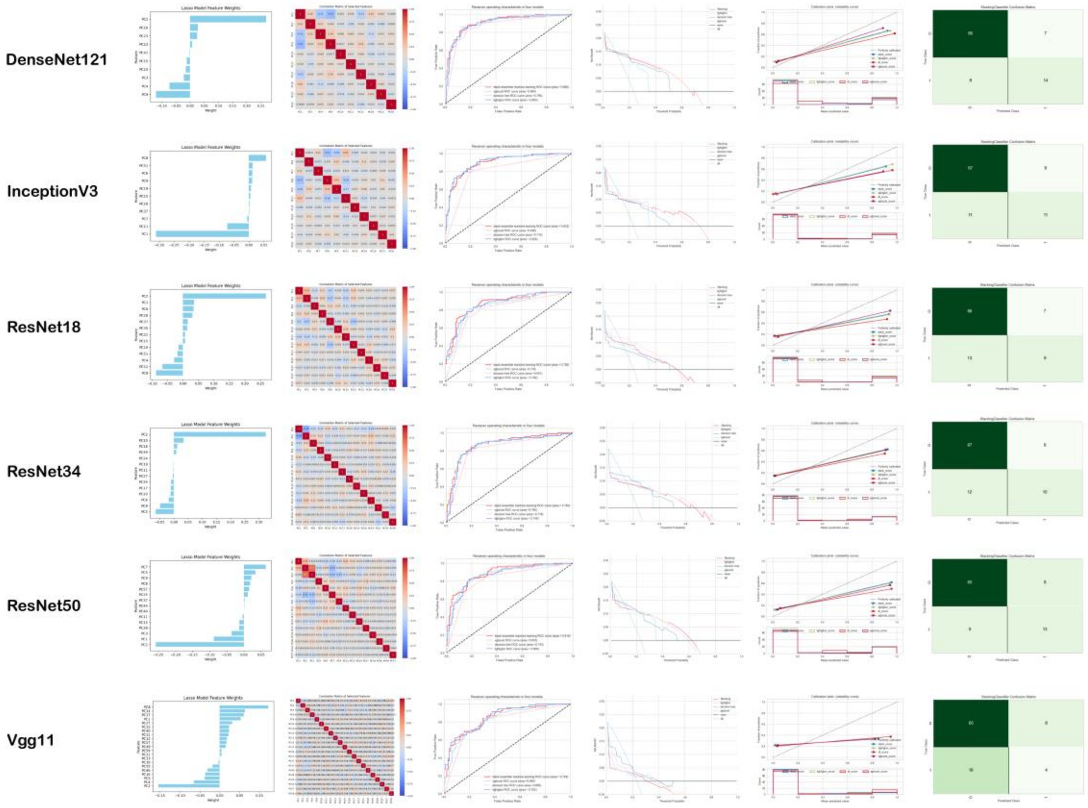

Figure S2 The result of six CNN feature-based stacking model and their base model(dt, xgboost, lightgbm) in Validation set

Table S2 Results of delong test for six CNN models

**Training set**	***p Value***	**Validation set**	***p Value***	**Testing set**	***p Value***
Densenet121	0.074	Densenet121	0.362	Densenet121	0.345
Inception_V3	0.005	Inception_V3	0.676	Inception_V3	0.631
ResNet18	0.031	ResNet18	0.273	ResNet18	0.181
ResNet34	0.135	ResNet34	0.161	ResNet34	0.573
ResNet50	reference	ResNet50	reference	ResNet50	reference
Vgg11	0.151	Vgg11	0.075	Vgg11	0.917

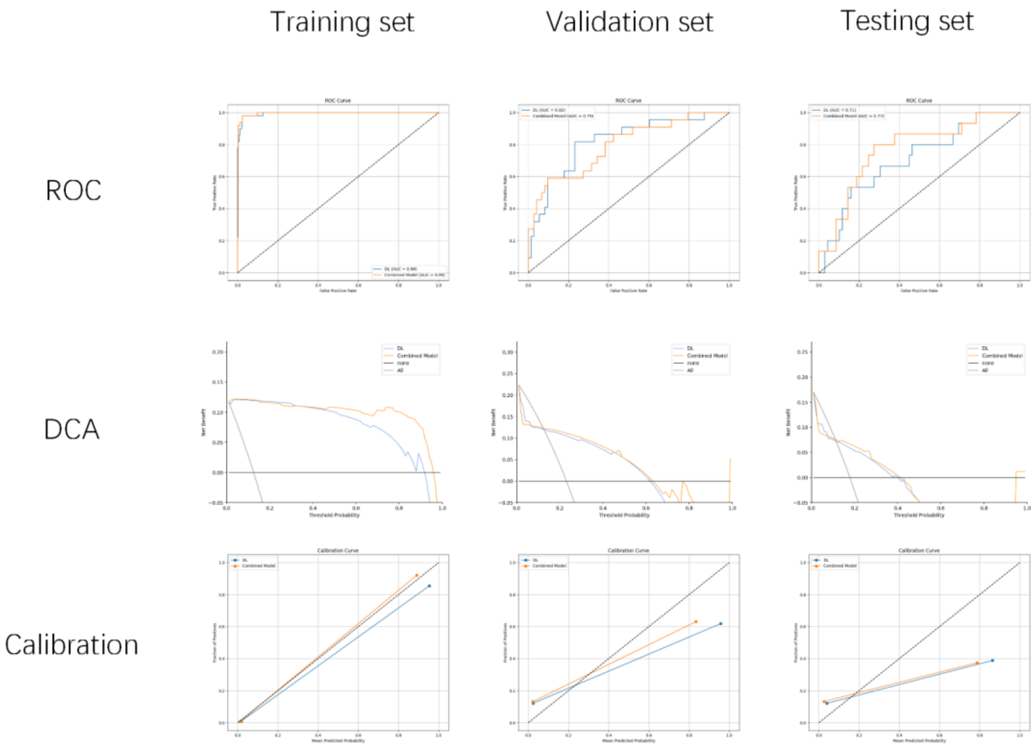

Figure S3 The result of DL Model and Combined Model (**with SMOTE**).

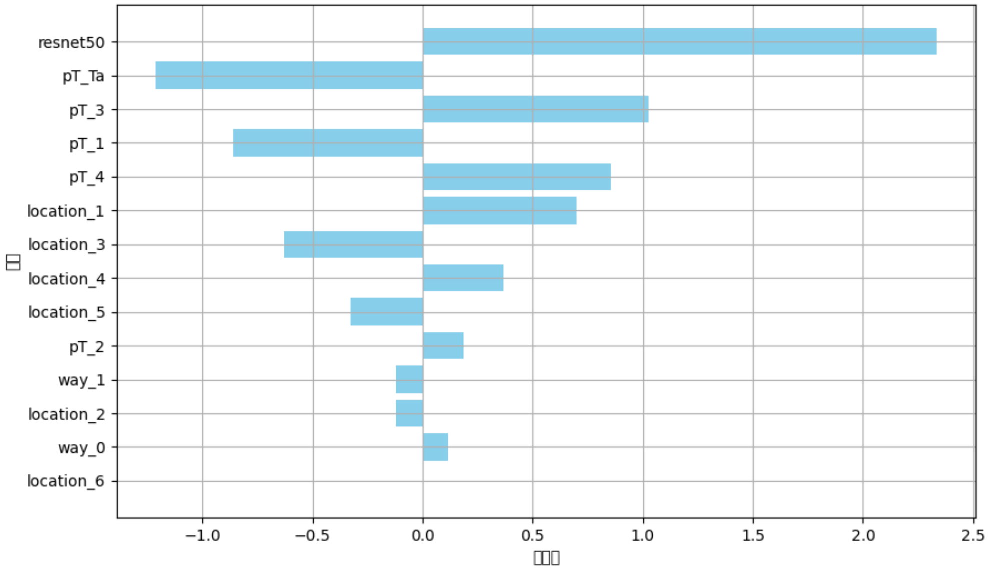

Figure S4 The coefficients of the factors in the Combined Model. ‘resnet50’=DL, ‘location_1’= left wall, ‘location_2’=right wall, ‘location_3’=anterior wall, ‘location_4’=posterior wall, ‘location_5’=parietal wall, ‘location_6’=bladder neck, ‘way_0’= within bladder wall, ‘way_1’= into bladder cavity

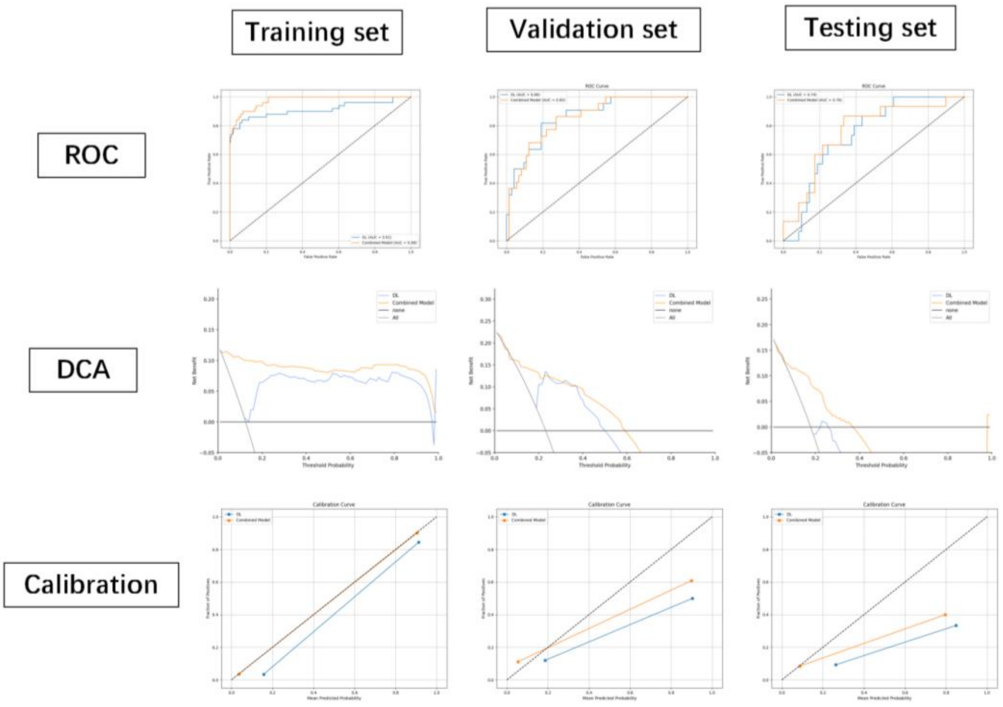

Figure S5 The result of original DL Model and original Combined Model (**without** SMOTE).

Table S3 The performance comparison of original CNN models (**without** SMOTE)

Model	Set	AUC	95%CI	ACC	95%CI	SEN	SPE
Densenet121	**Train**	0.909	0.844,0.973	0.957	0.957,0.957	0.780	0.983
	**Val**	0.796	0.670,0.921	0.789	0.786,0.793	0.818	0.781
	**Test**	0.658	0.493,0.823	0.750	0.746,0.754	0.533	0.797
Inception_V3	**Train**	0.913	0.848,0.977	0.927	0.927,0.927	0.820	0.943
	**Val**	0.819	0.712,0.927	0.800	0.797,0.803	0.773	0.808
	**Test**	0.668	0.508,0.827	0.631	0.626,0.636	0.800	0.594
ResNet18	**Train**	0.892	0.823,0.96	0.899	0.899,0.900	0.840	0.908
	**Val**	0.780	0.671,0.89	0.789	0.786,0.793	0.591	0.849
	**Test**	0.602	0.416,0.788	0.679	0.673,0.684	0.600	0.696
ResNet34	**Train**	0.976	0.959,0.994	0.912	0.911,0.912	0.980	0.902
	**Val**	0.800	0.689,0.91	0.842	0.839,0.845	0.682	0.890
	**Test**	0.713	0.587,0.839	0.583	0.578,0.589	0.933	0.507
ResNet50	**Train**	0.911	0.849,0.974	0.922	0.921,0.922	0.840	0.934
	**Val**	0.864	0.782,0.946	0.811	0.807,0.814	0.818	0.808
	**Test**	0.739	0.623,0.855	0.702	0.607,0.798	0.667	0.710
Vgg11	**Train**	0.804	0.721,0.887	0.864	0.864,0.865	0.660	0.894
	**Val**	0.699	0.566,0.831	0.758	0.754,0.762	0.545	0.822
	**Test**	0.680	0.521,0.839	0.631	0.626,0.636	0.800	0.594

CNN: Convolutional neural network; AUC: Area under the curve; ACC: Accuracy; SEN: Sensitivity; SPE: Specificity; 95%CI: Confidence Interval = 95%; Train: Training set; Val: Validation set; Test: Testing set

Table S4 The performance of models based on radiomics features

Model	Set	AUC	95%CI	ACC	95%CI	SEN	SPE
Radiomics	**Train**	0.827	0.752, 0.901	0.849	0.849, 0.850	0.760	0.862
	**Val**	0.740	0.612, 0.867	0.768	0.765, 0.772	0.636	0.808
	**Test**	0.705	0.545, 0.866	0.821	0.818, 0.825	0.533	0.884
Rad_smote	**Train**	0.981	0.969, 0.992	0.963	0.963, 0.963	0.986	0.940
	**Val**	0.804	0.731, 0.878	0.774	0.772, 0.776	0.767	0.781
	**Test**	0.714	0.628, 0.800	0.674	0.671, 0.677	0.696	0.652

Radiomics: The ensemble model based on radiomics features (without SMOTE); Rad_smote: The ensemble model based on radiomics features (with SMOTE); AUC: Area under the curve; ACC: Accuracy; SEN: Sensitivity; SPE: Specificity; 95%CI: Confidence Interval = 95%; Train: Training set; Val: Validation set; Test: Testing set

Table S5 The performance of clinical model

Model	AUC	95%CI	Accuracy	95%CI	Sens	Spec
Training set	0.843	(0.783, 0.897)	0.879	(0.847, 0.910)	0.1	0.991
Validation set	0.776	(0.667, 0.875)	0.768	(0.684, 0.853)	0.045	0.986
Testing set	0.665	(0.471, 0.855)	0.821	(0.738, 0.893)	0	1.000

AUC: Area under the curve; ACC: Accuracy; SEN: Sensitivity; SPE: Specificity; 95%CI: Confidence Interval = 95%; Train: Training set; Val: Validation set; Test: Testing set

CLEAR Checklist v1.0

Note: Use the checklist in conjunction with the main text for clarification of all items.
Yes, details provided; No, details not provided; n/e, not essential; n/a, not applicable; Page, page number

Section	No.	Item	Yes	No	n/a	Page
**Title**						
	1	Relevant title, specifying the radiomic methodology	✓			1
**Abstract**						
	2	Structured summary with relevant information	✓			1
**Keywords**						
	3	Relevant keywords for radiomics	✓			1
**Introduction**						
	4	Scientific or clinical background	✓			1
	5	Rationale for using a radiomic approach	✓			2
	6	Study objective(s)	✓			1-2
**Method**						
Study design	7	Adherence to guidelines or checklists (e.g., CLEAR checklist)	✓			2
	8	Ethical details (e.g., approval, consent, data protection)	✓			2
	9	Sample size calculation			✓	
	10	Study nature (e.g., retrospective, prospective)	✓			2
	11	Eligibility criteria	✓			2
	12	Flowchart for technical pipeline	✓			3
Data	13	Data source (e.g., private, public)	✓			2
	14	Data overlap		✓		
	15	Data split methodology	✓			2
	16	Imaging protocol (i.e., image acquisition and processing)	✓			2-3
	17	Definition of non-radiomic predictor variables			✓	
	18	Definition of the reference standard (i.e., outcome variable)	✓			4
Segmentation	19	Segmentation strategy	✓			2-3
	20	Details of operators performing segmentation	✓			2-3
Pre-processing	21	Image pre-processing details	✓			2-3
	22	Resampling method and its parameters	✓			2-3
	23	Discretization method and its parameters	✓			2-3

Section	No.	Item	Yes	No	n/a	Page
	24	Image types (e.g., original, filtered, transformed)	☑	☐	☐	2
Feature extraction	25	Feature extraction method	☑	☐	☐	3
	26	Feature classes	☐	☐	☑	
	27	Number of features	☑	☐	☐	3
	28	Default configuration statement for remaining parameters	☐	☐	☑	
Data preparation	29	Handling of missing data	☑	☐	☐	2
	30	Details of class imbalance	☑	☐	☐	3
	31	Details of segmentation reliability analysis	☐	☐	☑	
	32	Feature scaling details (e.g., normalization, standardization)	☑	☐	☐	3
	33	Dimension reduction details	☑	☐	☐	3
Modeling	34	Algorithm details	☑	☐	☐	3
	35	Training and tuning details	☑	☐	☐	3
	36	Handling of confounders	☑	☐	☐	3-4
	37	Model selection strategy	☑	☐	☐	3
Evaluation	38	Testing technique (e.g., internal, external)	☑	☐	☐	4
	39	Performance metrics and rationale for choosing	☑	☐	☐	4
	40	Uncertainty evaluation and measures (e.g., confidence intervals)	☑	☐	☐	3-4
	41	Statistical performance comparison (e.g., DeLong's test)	☑	☐	☐	3-4
	42	Comparison with non-radiomic and combined methods	☑	☐	☐	3-4
	43	Interpretability and explainability methods	☑	☐	☐	4
**Results**						
	44	Baseline demographic and clinical characteristics	☑	☐	☐	4
	45	Flowchart for eligibility criteria	☑	☐	☐	2
	46	Feature statistics (e.g., reproducibility, feature selection)	☐	☐	☑	
	47	Model performance evaluation	☑	☐	☐	4
	48	Comparison with non-radiomic and combined approaches	☑	☐	☐	4
**Discussion**						
	49	Overview of important findings	☑	☐	☐	4-5
	50	Previous works with differences from the current study	☑	☐	☐	4-5
	51	Practical implications	☑	☐	☐	5
	52	Strengths and limitations (e.g., bias and generalizability issues)	☑	☐	☐	5

Section	No.	Item	Yes	No	n/a	Page
**Open Science**						
Data availability	53	Sharing images along with segmentation data [n/e]	☐	☐	☑	
	54	Sharing radiomic feature data	☐	☐	☑	
Code availability	55	Sharing pre-processing scripts or settings	☑	☐	☐	appe
	56	Sharing source code for modeling	☑	☐	☐	appe
Model availability	57	Sharing final model files	☐	☐	☑	
	58	Sharing a ready-to-use system [n/e]	☐	☐	☑	

Kocak B, Baessler B, Bakas S, Cuocolo R, Fedorov A, Maier-Hein L, Mercaldo N, Müller H, Orlhac F, Pinto Dos Santos D, Stanzione A, Ugga L, Zwanenburg A. CheckList for EvaluAtion of Radiomics research (CLEAR): a step-by-step reporting guideline for authors and reviewers endorsed by ESR and EuSoMII. Insights Imaging. 2023 May 4;14(1):75. doi: 10.1186/s13244-023-01415-8
